# An Orthodenticle Homeobox 2 (OTX2) Mutation in a Patient With Combined Pituitary Hormone Deficiency, Pituitary Malformation, and Retinitis Pigmentosa

**DOI:** 10.7759/cureus.50819

**Published:** 2023-12-20

**Authors:** Cátia Araújo, Carla Baptista, Isabel Paiva

**Affiliations:** 1 Endocrinology, Diabetes and Metabolism, Centro Hospitalar e Universitário de Coimbra, Coimbra, PRT

**Keywords:** retinitis pigmentosa, otx2 mutation, neurodevelopment disorder, hypopituitarism, combined pituitary hormone deficiency

## Abstract

Heterozygous mutations of *orthodenticle homeobox 2 *(*OTX2*)can result in ocular malformations, pituitary abnormalities, or hypopituitarism spanning from isolated growth hormone (GH) deficiency to combined pituitary hormone deficiency. We present a patient exhibiting growth and pubertal disturbances, developmental delay, and pigmentary retinopathy. Further examination revealed deficiencies in GH following clonidine stimulation, hypogonadism, and, subsequently, central hypothyroidism. Brain magnetic resonance imaging uncovered hypoplasia of the pituitary and an ectopic pituitary tissue. Sequence analysis of *OTX2* identified a novel heterozygous mutation c.555_556dup, p.(Ser186Ilefs*21), indicative of a frameshift mutation. Replacement therapy with recombinant human GH, testosterone enanthate, and levothyroxine was started. Notably, GH therapy resulted in significant catch-up growth. This case report contributes to our comprehension of the molecular and clinical findings, particularly highlighting endocrine manifestations and a rare ophthalmologic manifestation associated with mutations in the *OTX2* gene.

## Introduction

Combined pituitary hormone deficiency (CPHD) is a genetically heterogeneous condition with various genes implicated in its pathogenesis, including *POU1F1, PROP1, LHX3, LHX4, HESX1, SOX2, SOX3, GLI2*, and *orthodenticle homeobox *2 (*OTX2*). The clinical presentation varies depending on age, as well as the number and severity of hormone deficiencies.

The *OTX2* gene plays a crucial role as it encodes a key transcription factor in the developmental processes of the brain, eyes, and craniofacial bones. Heterozygous mutations in OTX2 are linked to severe ocular phenotypes, brain malformations, and pituitary abnormalities. Recent findings include reports of early-onset retinal dystrophy associated with *OTX2* mutations, particularly retinitis pigmentosa [1-4].

Patients with *OTX2* mutations exhibit variable hypopituitarism, ranging from isolated growth hormone (GH) deficiency (IGHD) to CPHD, with GH deficiency being the most prevalent. Brain abnormalities may vary, presenting as a normal or hypoplastic anterior pituitary, a normal or ectopic posterior pituitary, and Chiari malformation. The incidence of pituitary hormone deficiencies in individuals with *OTX2* variants is reported to be 30% [5,6]. The severity of the disease not only varies based on specific *OTX2* mutations but also among individuals carrying the same mutation.

In this context, we describe a patient with *OTX2* mutation and CPHD, who additionally exhibited pituitary malformation, retinal dystrophy, and a neurodevelopment disorder.

## Case presentation

The patient is a 42-year-old male born to Caucasian nonconsanguineous parents at full term, with no further details available regarding birth history.

At the age of 16 years, he was referred to the pediatric endocrine unit because of short stature, delayed puberty, and neurodevelopmental disorder. He was prepubertal (Tanner I), height of 126.8 cm (-6.36 SDS), weight of 25 kg (-6.82 SDS), and body mass index of 15.5 (-2.07 SDS). His mid-parental target height was 162.6 cm. He exhibited a delayed bone age of 10 years. His eye structure was reported as being normal.

The pituitary function was assessed, and hormonal evaluations revealed low insulin-like growth factor 1 (IGF1), low insulin-like growth factor binding protein-3 (IGF-BP3), low total testosterone, low follicle-stimulating hormone (FSH), and low luteinizing hormone (LH) levels. GH stimulation tests with clonidine indicated abnormalities (1.9 ng/mL; expected > 7.4 ng/mL). Cortisol and adrenocorticotropic hormone (ACTH) levels, as well as thyroid stimulating hormone (TSH), total thyroxine (T4), and prolactin (PRL), were found to be normal at the time of diagnosis (Table 1).

**Table 1:** Initial laboratory results IGF1: insulin growth factor 1, IGF-BP3: insulin-like growth factor binding protein-3, FSH: follicle-stimulating hormone, LH: luteinizing hormone, TSH: thyroid-stimulating hormone, ACTH: adrenocorticotropic hormone, Total T4: total thyroxine, PRL: prolactin.

Parameters	Value	Reference range
IGF-1	20	223-903 ng/mL
IGF-BP3	0.4	3.4-9.5 µg/mL
Total testosterone	<1	2.6-15.9 ng/mL
FSH	0.16	1.5-14 mUI/mL
LH	<0.7	1.4-7.7 mUI/mL
ACTH	18.5	10-60 pg/mL
Cortisol	8	5-25 µg/dL
TSH	2.5	0.4-4 µUI/mL
Total T4	7.6	4.5-12.5 µg/mL
PRL	8	2.5-17 ng/mL

Magnetic resonance imaging of the brain revealed hypoplasia of the pituitary, an ectopic pituitary tissue, a normal pituitary stalk, and a small sella.

Moreover, the patient experienced night blindness and decreased peripheral vision and was diagnosed with retinitis pigmentosa. Notably, there was a significant family history of ocular abnormalities. The patient’s mother, two brothers, and one sister were also affected with retinitis pigmentosa. At the time, pituitary hormone deficiencies were not known or reported.

Puberty induction was started at the age of 16 with testosterone enanthate, initially at a dose of 125 mg every four weeks, later increased to 250 mg every four weeks. Recombinant GH therapy was initiated at the age of 16 years and six months with a dose of 0.63 U/kg/week, resulting in an improved growth rate one year later. GH therapy was discontinued at 19 years and 10 months, and the patient's final height was 163.9 cm.

At 18 years, additional laboratory findings have shown a low total T4 (3.7 µg/mL, reference range (RR): 4.5-12.5) and normal thyroid-stimulating hormone (TSH) (2,38 µUI/mL, RR: 0,4-4). Central hypothyroidism was diagnosed and levothyroxine treatment was initiated with 0.00125 mg/kg/day and later 0.0025 mg/kg/day. 

Genetic testing with whole-exome sequencing (WES), including copy number variation (CNV) analyses, was performed and identified a heterozygous duplication mutation NM_172337.2:c.555_556dup, p.(Ser186Ilefs*21) in exon 3 of *OTX2 *gene (Chr.14), considered a pathogenic variant.

## Discussion

We identified a novel mutation of the *OTX2* gene in a patient presenting with CPHD, retinal dystrophy, and intellectual disability. Specifically, a heterozygous mutation, c.555_556dup, p.(Ser186Ilefs*21) was detected. Given its nature (frameshift), both the introduction of a premature termination codon and subsequent production of a truncated protein are expected and can be considered pathogenic. This mutation has neither ever been described in the literature nor in gnomAD databases.

The variable presentation of clinical features and developmental course is associated with mutations in the *OTX2* gene. The relationship genotype/phenotype is complex, with incomplete penetrance observed in many families [7,8]. Genetic analysis is valuable for both diagnosis and genetic counseling.

GH deficiency is the predominant endocrinopathy in patients with this mutation, consistent with our case report. Although many patients experience growth or developmental retardation during childhood, most of them are born with weight and length within the normal range. GH therapy can enhance growth velocity, but there are wide variations in growth/clinical outcomes [9]. In our patient, therapy led to catch-up growth, resulting in a final height consistent with his mid-parental target height.

Additional hormone deficiencies may appear with age, as demonstrated by the central hypothyroidism seen in our patient. Therefore, regular evaluation of pituitary hormones is essential for early recognition of other hormone deficiencies.

Furthermore, developmental delay is often described, as seen in the patient. This may be linked to the *OTX2* mutations because *OTX2* is expressed in the brain and can be important for the development of the central nervous system.

Our patient was diagnosed with retinitis pigmentosa, a rare manifestation associated with *OTX2* mutation. The ophthalmological phenotype described in this case report aligns with the previous studies, demonstrating that mutations in this gene are linked to a wide range of phenotypes, with severe ocular malformations being more commonly described. Other family members were also found to have retinitis pigmentosa. Although these patients are highly clinically suspicious for harboring the same mutation, confirmation is pending, as we were unable to access genetic analyses of the siblings, and the patient’s mother declined genetic testing.

## Conclusions

This case adds further support for the role of *OTX2* gene both in retinitis pigmentosa and pituitary abnormalities and dysfunction, emphasizing a novel *OTX2* mutation. Genetic analysis is valuable for diagnosis and genetic counseling. GH deficiency is the most common pituitary hormone deficiency in these patients, and GH treatment showed efficacy in enhancing growth velocity. Therefore, a comprehensive clinical and biochemical follow-up is mandatory for early detection of pituitary hormone deficiency.
